# Pharmacokinetic study of metformin to compare a voglibose/metformin fixed-dose combination with coadministered voglibose and metformin 

**DOI:** 10.5414/CP202197

**Published:** 2014-12-29

**Authors:** Hyang-Ki Choi, Minkyung Oh, Eun Ji Kim, Geun Seog Song, Jong-lyul Ghim, Ji-Hong Shon, Ho-Sook Kim, Jae-Gook Shin

**Affiliations:** 1 Department of Pharmacology, Inje University College of Medicine, Busan,; 2 Clinical Development Division, CJ HealthCare Corp., Seoul, and; 3 Department of Clinical Pharmacology, Inje University Busan Paik Hospital, Busan, Republic of Korea

**Keywords:** pharmacokinetics, voglibose/metformin fixed-dose combination (FDC)

## Abstract

The aim of this study was to compare the pharmacokinetic characteristics of metformin between a fixed-dose combination (FDC) of voglibose/metformin and co-administered individual voglibose and metformin tablets in healthy Korean volunteers under fasting conditions. A randomized, open-label, single-dose, two-treatment, two-way crossover study with a 7-day wash-out period was conducted. Plasma samples were collected for up to 24 hours and were analyzed for metformin using a validated liquid chromatography tandem mass-spectrometry (LC/MS). A non-compartmental method was used to calculate the pharmacokinetic parameters. Vital signs and adverse events were monitored, and physical examinations and laboratory tests were conducted to evaluate safety. In total, 28 subjects completed the study. The geometric mean ratio (GMR) and the 90% confidence interval (CIs) of C_max_ and AUC_0–t_ of metformin were 102.4 (94.5 – 111.0) and 107.1 (100.1 – 114.7), respectively. In total, 7 adverse drug reactions occurred in 4 subjects during the study; of these, 3 cases were from 3 subjects in the test treatment group, and 4 cases were from 3 subjects in the reference treatment group. All adverse drug reactions had been reported previously, and all subjects recovered fully without any sequelae. In conclusion, the pharmacokinetic profiles of metformin in two different study treatments, a voglibose/metformin FDC vs. the coadministration of the individual formulations, met the regulatory criteria for bioequivalence in healthy Korean subjects under fasting conditions. There was no significant difference in safety profiles between the two treatments.

Clinical Trial Registration

ClinicalTrials.gov number, NCT01370681. Available at URL. http://clinicaltrial.gov/

## Introduction 

Diabetes mellitus (DM) is a disease of metabolic dysregulation, characterized by chronic hyperglycemia due to insufficient insulin action [1, 2]. The prevalence of diabetes has been increasing continuously. It has been reported that ~ 382 million people have diabetes worldwide in 2013, and it will rise to 592 million by 2035 [3]. Diabetes is associated with various complications, such as diabetic foot neuropathy, diabetic nephropathy, diabetic retinopathy, cataracts, and glaucoma [4, 5, 6], which cause more than 300,000 deaths annually worldwide [2, 7]. To prevent serious and long-term complications and reduce mortality from diabetes, continuous treatment and patient education is necessary [8]. 

Metformin, an oral antihyperglycemic agent, is extensively used in the treatment of type 2 diabetes. Metformin reduces gluconeogenesis in the liver by activating AMP-activated protein kinase (AMPK) via liver kinase B1 (LKB1) [9]. It also increases glucose utilization and insulin sensitivity in peripheral tissues including muscles and fat. As a result, metformin lowers plasma glucose level, in the fasting condition [10, 11, 12, 13]. Additionally, it reduces triglycerides and low-density lipoprotein (LDL)-cholesterol, which is helpful in maintaining a favorable cholesterol profile [14, 15]. 

Voglibose, an α-glucosidase inhibitor, is also used widely in the management of type 2 diabetes. It undergoes minimal systemic absorption [16]. Voglibose delays the absorption of carbohydrates due to competitive inhibition of α-glycosidase in the small intestine [17, 18, 19]. Consequently, voglibose inhibits the postprandial increase in plasma glucose levels, leading to decreased diurnal insulin secretion [17, 20]. 

According to the American Diabetes Association guideline, metformin is suggested as the preferred initial agent, and, if metformin monotherapy fails to reduce or maintain blood glucose, other therapeutic agents should be added to metformin [21]. Fixed-dose combinations (FDCs) of metformin and other diabetes drugs, such as metformin + sulfonylurea (glibenclamide, glipizide, gliclazide glimepiride), metformin + glinide (repaglinide), and metformin + DPP-4 inhibitor (sitagliptin, saxagliptin, vildagliptin), have been developed to improve convenience and compliance with multiple medications [22]. Recently, a FDC of voglibose and metformin, which can increase the patient drug compliance while lowering the side effect of hypoglycemia, was developed. Combining the benefits of the two different mechanisms of action, a FDC of voglibose and metformin is intended to provide an intensive initial blood glucose management regimen in newly diagnosed diabetic patients by simultaneously regulating the fasting as well as the postprandial blood glucose, and thereby delaying the progression of the disease. 

The objective of this study was to evaluate the bioequivalence of metformin administered as a single dose of a voglibose/metformin 0.2/500 mg FDC tablet vs. the co-administration of voglibose 0.2 mg and metformin 500 mg in healthy Korean volunteers under fasting conditions. Only the pharmacokinetics of metformin were evaluated in this study because voglibose is undetectable after therapeutic dosing [16]. 

## Methods 

### Subjects 

Healthy volunteers, aged 20 – 55 years, who were within 20% of their standard body weight according to the Broca formula, were considered for participation. Subjects who had not experienced congenital or chronic disease, were judged to be healthy based on the results of a detailed clinical examination, could participate in the whole clinical trial, and voluntarily signed a written informed consent form were eligible for inclusion in this study. The following exclusion criteria were used: administration of inducers or inhibitors of drug-metabolizing enzymes within 1 month, symptoms of acute disease within 4 weeks, a history of allergic disease or hypersensitivity reactions, abnormal laboratory test results, excessive consumption of caffeine (> 5 cups/day), cigarettes (> 10 cigarettes/day), or alcohol (> 30 g of alcohol/day), a diet containing foods known to affect the absorption, distribution, metabolism, and excretion of the study drugs (e.g., grapefruit juice), a history of participation in another clinical study within 90 days, a whole-blood donation within 60 days, pregnant/lactating females, and females of childbearing potential not practicing a medically acceptable method of contraception. 

### Study design 

The study protocol was approved by the Inje University Busan Paik Hospital Institutional Review Board (IRB No: 09-117). The study was carried out in compliance with the Declaration of Helsinki, the International Conference on Harmonisation of Good Clinical Practice (ICH-GCP), and the current Korean Good Clinical Practice (KGCP) guidelines. 

The clinical trial was conducted at the Inje University Busan Paik Hospital clinical trial center, Busan, Korea, from October 29, 2009 to November 17, 2009 (ClinicalTrials.gov identifier: NCT01370681). All participants were informed of the study objectives, potential risks, and compensation before joining the study. All eligible subjects provided written informed consent to participating and were free to withdraw from the study at any time without obligation. Voglibose/metformin 0.2/500 mg FDC (CJ HealthCare Corp., Seoul, Republic of Korea) was used as the test treatment, and the coadministration of voglibose 0.2 mg (Basen, CJ HealthCare Corp., Seoul, Republic of Korea) and metformin 500 mg (Glucophage, Merck, West Drayton, UK) were used as the reference treatment. 

This was an open-label, randomized, single-dose, two-way crossover study in healthy male subjects. Subjects were allocated to each group (RT, TR) in a 1 : 1 ratio according to a predesigned randomization table that was generated using SAS software (ver. 9.2: SAS Institute Inc., Cary, NC, USA). There was a screening period of up to 28 days prior to study drug administration on day 1. Subjects received one of the two treatments according to the group they were allocated to. After a 7-day wash-out period, which was determined to be more than seven times the half-life of metformin [23] and convenient for the study, each subject received the other treatment. 

After an overnight fast prior to study drug administration on day 1, the study drug was administered with 240 mL tap water, supervised by physicians. An oral check was performed immediately after the administration in each subject to ensure compliance. Water was permitted 2 hours after the study drug administration and food 4 hours after. 

For the pharmacokinetic analysis of metformin, blood samples were collected at pre-dose (0 hour) and 0.5, 1, 1.5, 2, 2.5, 3, 3.5, 4, 6, 8, 10, 12, and 24 hours after administration in each period. Whole blood samples (7 mL) were collected in heparinized vacutainer tubes, and then centrifuged (2,000 g, 10 minutes) to separate plasma. Plasma samples (1 mL) were transferred to microcentrifuge tubes and stored at –80 °C until analysis. 

### Safety assessment 

Subjects who received at least one treatment during the study were included in the safety assessment analysis. To evaluate safety, vital signs (including sitting blood pressure, heart rate, and tympanic temperatures), physical examination, and laboratory tests (including hematology, biochemistry, and urinalysis) were assessed. Vital signs were measured before (0 hours), and at 3, 12, and 24 hours after administration in each period. Physical examination and laboratory tests were evaluated at the time of hospitalization and discharge. 

### Bioanalysis 

Metformin concentrations in plasma were analyzed using liquid chromatography tandem mass spectrometry (Agilent 1200 series HPLC and Agilent 6410 LC-MS/MS system; Agilent Technologies, Santa Clara, CA, USA), based on a method developed previously [24]. Metformin and an internal standard (propranolol) were dissolved in methanol. Detection and quantification were performed using a triple quadrupole tandem mass spectrometer with an electrospray ionization interface in positive mode and multiple-reaction-monitoring mode. Chromatographic separation of the compounds was accomplished using a synergic Polar-RP column (4 µm, 2.0 × 150 mm; Phenomenex, Torrance, CA, USA) with 55% acetonitrile in water containing 0.1% formic acid as the mobile phase, and a flow rate of 0.2 mL/min. 

Briefly, 500 µL of acetonitrile containing the internal standard (propranolol, 50 ng/mL) were added to 100 µL of plasma sample. After vortex-mixing for 10 minutes and centrifugation (16,000 g, 10 minutes), the supernatant was injected into the LC-MS/MS system. A full validation of the assay was carried out with respect to selectivity, accuracy, precision, calibration curve, and stability. The calibration curve of metformin was linear over the range of 10 – 2,000 ng/mL, and the lowest limit of quantification was 10 ng/mL, with a coefficient of determination (R^2^) greater than 0.994. The intra- and interday precision was 1.70 – 5.31 and 4.44 – 11.33%, respectively. The intra- and interday accuracy was 97.31 – 112.01% and 100.94 – 106.95%, respectively. There was no significant interference in selectivity or stability. 

### Pharmacokinetic and statistical analyses 

The Korean bioequivalence study guideline (the version when conducting this study) recommends that bioequivalence studies should be conducted with at least 12 subjects per group (total, 24 subjects) [25]. 30 subjects are needed with an expected dropout rate of 20%, based on dropout rates during bioequivalence studies at our clinical trial center. The sample size, based on an inter-subject coefficient of variation of 22% for both the AUC_0–t_ and C_max_ of metformin [23], was calculated as 9 subjects per group to detect a 20% difference between the test and reference treatments with power of 80% at a significance level of 5%. Thus, 30 subjects were considered to be sufficient to evaluate the bioequivalence of metformin. 

The pharmacokinetic parameters of metformin were estimated using a noncompartmental method with WinNonlin software (ver. 5.3; Pharsight Corporation, Mountain View, CA, USA). The maximum concentration (C_max_) and area under the concentration-time curve from zero (predose) to time of last quantifiable concentration (AUC_0–t_) were the primary parameters, and time to reach C_max_ (t_max_), half-life (t_1/2_), and AUC_0–∞_ were estimated as secondary parameters. C_max_ and t_max_ were obtained from the observed plasma concentration-time profile. AUC_0–t_ was calculated according to the linear trapezoidal rule. AUC_0–∞_ was calculated with the following equation: AUC_0–∞_ = AUC_0–t_ + C_t_/λ_z_, where C_t_ is the last observed concentration and λ_z_ is the elimination rate constant. The t_1/2_ was calculated as 0.693/λ_z_. Subjects who completed the study were included in the pharmacokinetic analysis. 

For the bioequivalence test, the geometric mean ratios of C_max_ and AUC_0–t_ of metformin were calculated. If the 90% confidence intervals (CIs) of the geometric mean ratio of primary parameters were within the range 0.8 to 1.25, the new formulation was considered to meet the criteria for bioequivalence [26, 27]. A mixed-effects analysis of variance (ANOVA) model was performed on the log-transformed C_max_ and AUC with random effects of sequence-nested subject, and fixed effects of sequence, period, and treatment. All statistical analyses were performed using the SAS software (ver. 9.2; SAS Institute Inc., Cary, NC, USA). A p-value of < 0.05 was considered to indicate statistical significance. 

## Results 

### Study subjects 

In total, 30 healthy subjects were enrolled. Two subjects dropped out before the first administration of the investigational product because of laboratory test abnormalities. Thus, 28 subjects participated from the beginning of the study and completed the study. They were included in the pharmacokinetic analysis and safety assessment. Their mean (standard deviation, SD) age, height, and weight were 23.97 (1.56) years, 174.67 (6.10) cm, and 72.03 (8.27) kg, respectively. 

### Pharmacokinetics 

**Figure 1.**
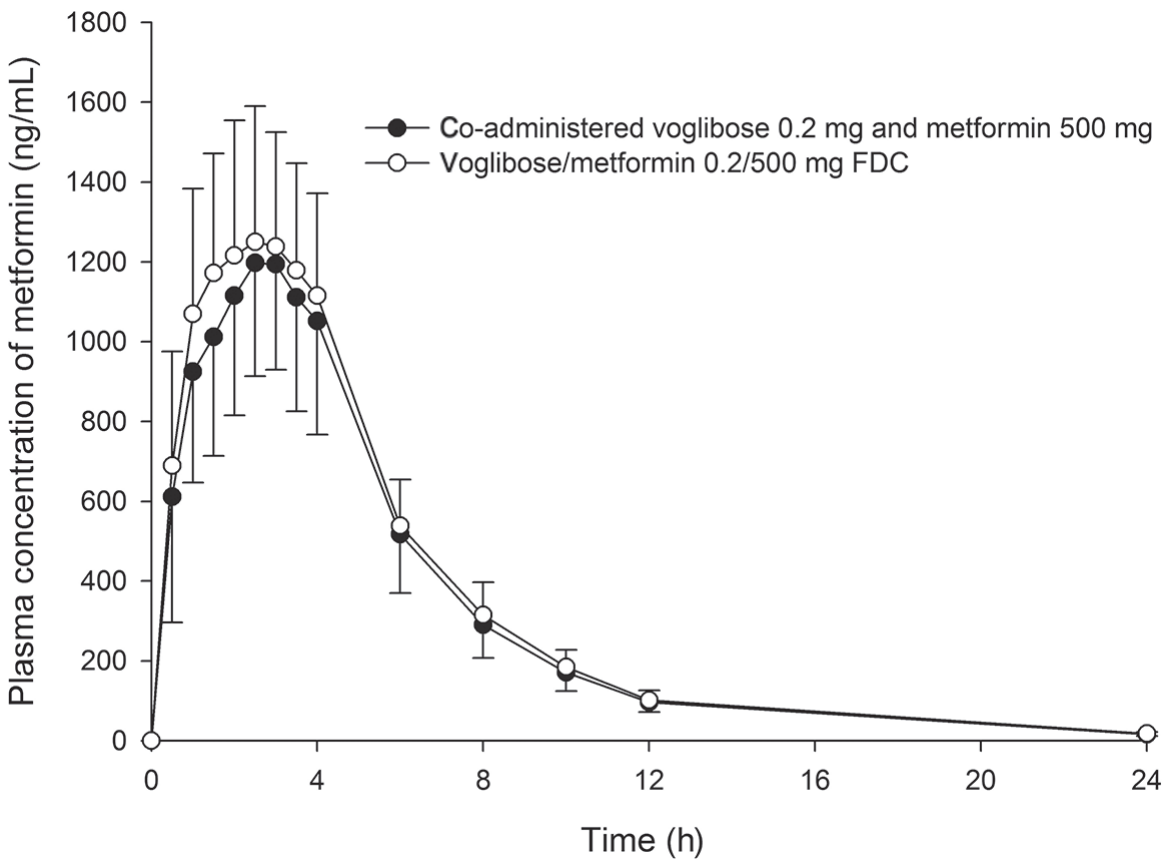
Mean plasma concentration-time curve of metformin following a single oral administration of voglibose/metformin 0.2/500 mg fixed-dose combination (FDC) (open circle) vs. coadministered voglibose 0.2 mg and metformin 500 mg (closed circle) in 28 healthy subjects.

Mean plasma concentration-time curves of metformin after single-dose oral administration of the test and reference treatments are shown in Figure 1. The mean (SD) C_max_ values of metformin after administration of the test and reference treatments were 1.38 (0.32) and 1.35 (0.30) µg/mL, respectively. The mean (SD) AUC_0–t_ and AUC_0–∞_ values were 8.17 (1.65) and 8.28 (1.65) µg×h/mL, respectively, after administration of the test treatment. The mean (SD) AUC_0–t_ and AUC_0–∞_ values were 7.63 (1.54) and 7.73 (1.54) µg×h/mL, respectively, after administration of the reference treatment (Table 1). No statistically significant difference in t_max_ or t_1/2_ was observed between the test and reference treatments. 

The geometric mean ratio and its CIs for metformin are presented in Table 2. The 90% CIs for the ratio for C_max_ and AUC_0–t_ of metformin were 94.5 – 111.0% and 100.1 – 114.7%, respectively. 

### Safety 

No serious adverse event occurred during the study. Six of the 28 subjects experienced adverse events during the study; 4 subjects showed adverse drug reactions, such as loose stools and epigastric discomfort, after administration of both the test and reference; 1 subject showed loose stool after coadministration of voglibose and metformin, 2 subjects had loose stool after both treatments, and the other experienced loose stool and epigastric discomfort after administration of FDC and after coadministration of individual tablets. Periodontitis and fever were considered unlikely to be drug-related following a causality assessment. All adverse events were classified as being mild-to-moderate in severity. All subjects recovered fully without any sequelae. No clinically significant change was seen in the laboratory tests results, including hematology, biochemistry, and urinalysis, vital signs, or physical examination. No significant difference was observed in adverse events or adverse drug reactions between the test and reference treatments (Table 3). 

## Discussion 

In the present study, we compared the pharmacokinetics of metformin and the safety profiles between a newly developed FDC tablet containing voglibose/metformin 0.2/500 mg and the coadministration of individual voglibose 0.2 mg and metformin 500 mg formulations in healthy Korean volunteers. This is the first study on the bioequivalence of metformin for the voglibose/metformin FDC tablet. The 90% CIs for the geometric mean ratio for C_max_ and AUC_0–t_ of metformin fell entirely within the regulatory criteria for bioequivalence: The acceptable range is 80 – 125% according to the current US Food and Drug Administration guidelines [26]. The results demonstrated that metformin was bioequivalent when compared with the test drug (voglibose/metformin FDC) and the reference drug (coadministered voglibose and metformin). 

In present study, the C_max_ of metformin were calculated to 1.38 µg/mL and 1.35 µg/mL for voglibose/metformin FDC tablet and the coadministration of individual voglibose and metformin, respectively. The AUC_0–t_ values were 8.17 µg×h/mL and 7.63 µg×h/mL for the FDC tablet and coadministration of individual formulations, respectively. These values of pharmacokinetic parameters were similar to prior metformin pharmacokinetic study results [28]. These comparable results suggest that bioanalytical and pharmacokinetical analysis had been appropriately performed in this study. 

There are several factors that affect the pharmacokinetics of metformin. Food delays metformin absorption and decreases C_max_ and AUC of metformin by ~ 40% and 25%, respectively [29]. The study drug in this study is not a modified release formulation, and, thus, it is expected that food haa a similar effect on the metformin pharmacokinetics of this FDC tablet. Metformin is excreted unchanged in the urine and does not undergo metabolism [30]; therefore, decreased renal function reduces the metformin renal clearance, leading to increased C_max_ and AUC. However, this factor seems not to have influence on the results of bioequivalence. 

Because voglibose is poorly absorbed in the gastrointestinal tract, its concentration in plasma and urine is not practically detectable in the clinical situation [16]; therefore, only the pharmacokinetics of metformin were compared in this study. Administration of sucrose is needed to understand the pharmacodynamic characteristics of voglibose [31]. However, this is not compatible with fasting, and it is therefore not possible to perform a traditional pharmacokinetic bioequivalence study. A pharmacodynamic study of voglibose is in progress to evaluate the pharmacodynamic equivalence of the voglibose/metformin FDC vs. coadministered individual formulations. 

No serious adverse event occurred in the present study. All adverse events were mild and resolved without specific intervention or sequelae and were in line with those known for the reference drug [23]. The incidence of adverse drug reactions was similar between the voglibose/metformin FDC and the coadministration of the two individual formulations. This indicates that the voglibose/metformin 0.2/500 mg FDC is likely to have a similar safety profile as the reference treatment. The most frequently observed adverse drug reactions, in the current study 14% (4 of 28 subjects) of subjects, were gastrointestinal disorders including diarrhea, and epigastric discomfort, which are well known as common responses after metformin administration. A previously published metformin efficacy study in patients [10] reported that digestive disturbances occurred in 22% (16 of 73 subjects) of subjects. Our previous drug-drug interaction study between voglibose and metformin [32] also reported that 33% (8 of 24 subjects) of subjects experienced gastrointestinal symptoms following metformin 500 mg multiple administration. When compared to these prior studies, the present study showed a relatively low incidence of gastrointestinal disorder, possibly because this study was designed as a metformin single oral administration study. 

## Conclusions 

Metformin of the voglibose/metformin FDC met the regulatory criteria for bioequivalence compared to coadministered individual voglibose and metformin. Both the FDC formulation and individual tablets were well tolerated and their safety profiles were not significantly different. 

## Acknowledgments 

This study was sponsored by CJ HealthCare Corp., Republic of Korea and supported partly by a grant of the Korea Health Technology R&D Project through the Korea Health Industry Development Institute (KHIDI), funded by the Ministry of Health & Welfare, Republic of Korea (HI14C1063). We thank Su-Jin Park and Ji-Eun Seo for their support in this clinical trial. 

## Conflict of interest 

Eun Ji Kim and Geun Seog Song are full-time employees of Clinical Development Division, CJ HealthCare Corp. The other authors have no conflicts of interest to disclose. 

**Table 1.** Pharmacokinetic parameters of metformin following a single administration of voglibose/metformin 0.2/500 mg fixed-dose combination (FDC) vs. co-administered voglibose 0.2 mg and metformin 500 mg in 28 healthy subjects.

Parameter	Voglibose/metformin FDC	Coadministered voglibose and metformin
C_max_, (µg/mL)	1.38 (1.35) ± 0.32	1.35 (1.32) ± 0.30
AUC_0–t_, (µg×h/mL)	8.17 (8.01) ± 1.65	7.63 (7.48) ± 1.54
AUC_0–∞_, (µg×h/mL)	8.28 (8.13) ± 1.65	7.73 (7.58) ± 1.54
t_max_, (h)	2.5 (1 ~ 4)	2.5 (0.5 ~ 4)
t_1/2_, (h)	4.08 ± 0.62	4.20 ± 0.69

All values are arithmetic mean (geometric mean) ± standard deviation except t_max_ is median (range).

**Table 2.** Geometric mean ratio (GMR) and the 90% CIs for metformin.

Parameter		GMR^†^ (%)	90% CIs
Metformin	C_max_ (µg/mL)	102.4	94.5 – 111.0
	AUC_0–t_ (µg×h/mL)	107.1	100.1 – 114.7
	AUC_0–∞_ (µg×h/mL)	107.2	100.3 – 114.6

^†^GMR represents geometric mean ratio of voglibose/metformin fixed-dose combination (test) to coadministered voglibose and metformin (reference) (test/reference). These estimates are obtained using least square means from the mixed effects model with the fixed effects for sequence, period and treatment, and a random effect for subjects within sequence.

**Table 3.** Adverse events observed during the study of voglibose/metformin fixed-dose combination (FDC) and coadministered metformin and voglibose in healthy Korean subjects.

System organ class Preferred term	Voglibose/metformin FDC^†^ (n = 28)	Co-administered voglibose and metformin^† ^ (n = 28)
Gastrointestinal disorders		
Loose stools	3 (3)^‡^	3 (3)^‡^
Epigastric discomfort		1 (1)^‡^
Periodontitis	1 (1)	
Body as a whole/general disorders		
Fever	1 (1)	
Total (adverse events)	5 (5)	4 (3)
Total (adverse drug reactions)	3 (3)	4 (3)

^†^Data represents number of cases (number of subjects); ^‡^Considered to be study drug-related.
